# Unusual Case of Left Testicular Pain Due to Pampiniform Venous Plexus Thrombosis: A Case Report

**DOI:** 10.7759/cureus.51044

**Published:** 2023-12-24

**Authors:** Abdullah Ayed Alshahrani

**Affiliations:** 1 Department of Surgery, University of Bisha, Bisha, SAU

**Keywords:** urology trauma, scrotal doppler ultrasound, venous plexus thrombosis, testicular pain, venous plexus, pampiniform plexus

## Abstract

Spontaneous thrombosis of the pampiniform venous plexus is a rare phenomenon, with limited cases reported in the literature. Accurate diagnosis necessitates a high index of suspicion, and scrotal Doppler ultrasound proves to be an effective diagnostic tool. This case report aims to elucidate the diagnostic intricacies of spontaneous thrombosis of the left pampiniform venous plexus, emphasizing the pivotal role of scrotal Doppler ultrasound. Additionally, we explore successful treatment modalities, including anticoagulation and bed rest, leading to complete resolution. This contribution aims to enhance clinical understanding, particularly in outpatient and emergency care settings, where accurate and timely diagnosis is imperative.

## Introduction

Spontaneous thrombosis of the pampiniform venous plexus is an infrequent occurrence, with only a handful of cases documented in the medical literature [1]. While it can manifest in individuals across age groups, its rarity and potential to mimic other causes of acute scrotal pain, such as testicular torsion or epididymal-orchitis, pose diagnostic challenges [2,3]. This case report sheds light on the diagnostic intricacies of spontaneous thrombosis of the left pampiniform venous plexus, emphasizing the significance of heightened clinical suspicion, particularly in cases of acute scrotal pain.

Accurate and timely diagnosis is crucial, as some instances have been unveiled only during testicular exploration prompted by severe pain, raising concerns about the potential oversight of testicular torsion [4]. The diagnostic approach involves employing scrotal Doppler ultrasound, a reliable tool that can reveal characteristic features of thrombosis, including the presence of echogenic particles, non-compressibility, and cessation of blood flow [5,6]. While computed tomography scans and CT angiography can also assist in diagnosis [1], our focus is on highlighting the pivotal role of scrotal Doppler ultrasound in providing a non-invasive and effective means of confirming pampiniform venous plexus thrombosis.

Furthermore, this case report aims to contribute valuable insights to clinical practice. The observed left testicular pain in our patient and subsequent successful management through anticoagulation and bed rest underscore the importance of considering spontaneous thrombosis of the pampiniform venous plexus in the differential diagnosis of acute scrotal pain. Such awareness is particularly relevant in outpatient clinics, where urological evaluations commonly occur, and in the emergency room setting, where prompt and accurate diagnosis can prevent unnecessary surgical interventions. This report advocates for a comprehensive understanding of this rare condition to guide clinicians in providing optimal care and avoiding potential misdiagnoses, thereby advancing clinical practice in both outpatient and emergency care settings.

## Case presentation

A 23-year-old male patient with no history of any chronic medical illnesses presented to the ER of our hospital complaining of left testicular pain for seven days. The pain was sudden in onset, gradually increasing in severity, and associated with mild left inguinal pain and swelling. No history of lower urinary tract symptoms, fever, or urethral discharge. The patient was recently married two months earlier and had a history of vigorous sexual intercourse but no prior history of sexual contact. There was no vomiting, abdominal pain, constipation, or history of a chronic cough. No history of hematuria, loin pain, or loin swelling. The patient has had left inguinal hernia repair since childhood.

On physical examination, the patient had some tenderness around the left inguinal region with some induration and left grade 3 varicocele. The left testis was tender, otherwise normal. There was no abdominal distention or palpable abdominal masses, and there was no left loin pain or mass.

**Figure 1:**
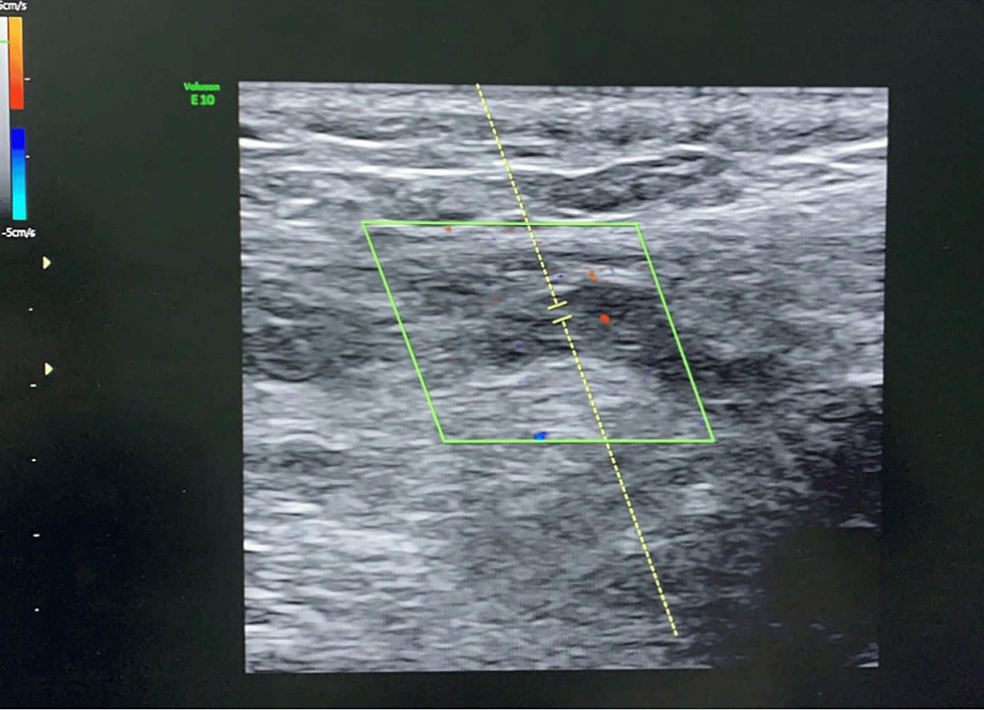
Left scrotal Doppler ultrasound revealed a dilated vein in the left spermatic cord.

A Doppler ultrasound of the scrotum showed a swollen vein in the left spermatic cord that was filled with echogenic particles (thrombus) (Figure 1). The pictures were consistent with a thrombosed left pampiniform venous plexus. The abdominal ultrasound scan was normal. 

The patient was admitted to the ward for bed rest; anti-inflammatory medication, analgesics, and a curative dose of anticoagulant were administered. He showed remarkable improvement over a period of seven to 10 days and was discharged home. The patient was followed up in the outpatient clinic, and two months later, there was a complete resolution of the thrombus with only the persistence of the varicocele.

## Discussion

Thrombosis of a varicocele is primarily managed conservatively in most reported cases, with only a minority undergoing surgical intervention [2]. The literature reveals the rarity of spontaneous thrombosis in the pampiniform venous plexus, posing diagnostic challenges and often leading to misdiagnoses such as incarcerated inguinal hernia or other causes of acute scrotal pain [4]. The etiologies of spontaneous thrombosis in the left spermatic vein remain unknown, with hypotheses including Buerger's illness and prolonged sexual activity [6].

Our findings resonate with the broader literature, emphasizing the infrequency of pampiniform venous plexus thrombosis and its potential to mimic other scrotal pathologies. Diagnostic challenges highlighted in our case, particularly the need for a high index of suspicion, align with observations from previous cases [2,4].

Doppler ultrasound of the superficial pampiniform plexus serves as a confirmatory diagnostic tool, revealing hypoechoic, non-compressible, cystic inguinal masses with absent blood flow, especially when thrombosis is limited to the superficial plexus [6]. Despite the lack of fixed guidelines due to the rarity of cases, the literature review emphasizes conservative management with non-steroidal anti-inflammatory drugs, anticoagulants, and bed rest [6].

The clinical relevance of our findings extends to outpatient and emergency care settings, stressing the importance of heightened awareness for accurate and timely diagnosis, thereby averting unnecessary surgical interventions. Successful conservative management, in our case, aligns with established practices [6].

While surgical intervention is rare, instances have been documented, emphasizing the need for cautious consideration and preoperative diagnosis to avoid unnecessary interventions. This highlights the significance of distinguishing pampiniform venous plexus thrombosis from other surgical emergencies.

Acknowledging the limitations inherent in a single-case report and the overall rarity of this condition, our findings underscore the necessity for continued research. Future investigations should focus on larger cohorts to validate diagnostic and management approaches. Additionally, efforts should be directed toward establishing standardized protocols for diagnosing and managing pampiniform venous plexus thrombosis, ensuring consistency in clinical practices.

## Conclusions

In summary, pampiniform venous plexus thrombosis diagnosis relies on Doppler imaging, and due to its rarity, conservative management is the predominant approach. Additional research is warranted to develop standardized protocols for the diagnosis and management of this condition. This case report not only enhances our comprehension of a rare condition but also underscores the significance of scrotal Doppler ultrasound in diagnosis, emphasizing its potential influence on clinical practices and advocating for increased awareness among clinicians in both outpatient and emergency care settings, promoting accurate and timely diagnosis.
